# Eyes on AI: ChatGPT's Transformative Potential Impact on Ophthalmology

**DOI:** 10.7759/cureus.40765

**Published:** 2023-06-21

**Authors:** Jason Dossantos, Jella An, Ramin Javan

**Affiliations:** 1 Ophthalmology, George Washington University School of Medicine and Health Sciences, Washington, USA; 2 Ophthalmology, Johns Hopkins University School of Medicine Wilmer Eye Institute, Baltimore, USA; 3 Radiology, George Washington University School of Medicine and Health Sciences, Washington, USA

**Keywords:** artificial intelligence in medicine, large language models, artificial intelligence, chatgpt, ophthalmology

## Abstract

ChatGPT, a large language model by OpenAI, has been adopted in various domains since its release in November 2022, but its application in ophthalmology remains less explored. This editorial assesses ChatGPT's potential applications and limitations in ophthalmology across clinical, educational, and research settings. In clinical settings, ChatGPT can serve as an assistant, offering diagnostic and therapeutic suggestions based on patient data and assisting in patient triage. However, its tendencies to generate inaccurate results and its inability to keep up with recent medical guidelines render it unsuitable for standalone clinical decision-making. Data security and compliance with the Health Insurance Portability and Accountability Act (HIPAA) also pose concerns, given ChatGPT’s potential to inadvertently expose sensitive patient information. In education, ChatGPT can generate practice questions, provide explanations, and create patient education materials. However, its performance in answering domain-specific questions is suboptimal. In research, ChatGPT can facilitate literature reviews, data analysis, manuscript development, and peer review, but issues of accuracy, bias, and ethics need careful consideration. Ultimately, ensuring accuracy, ethical integrity, and data privacy is essential when integrating ChatGPT into ophthalmology.

## Editorial

Introduction

ChatGPT is a large language model (LLM) developed by OpenAI that can generate natural language texts based on user inputs [1]. Since its release in November 2022, it has been widely adopted, reaching 100 million users in two months and has shown to produce impressive results in various domains [1]. The application of ChatGPT has been explored in several fields, such as interventional radiology and otolaryngology [2,3]. However, its utilization in ophthalmology has been limited. In this paper, we will propose potential applications and limitations of ChatGPT in ophthalmology.

Clinical applications and limitations

ChatGPT can act as a clinical assistant to provide diagnostic or therapeutic suggestions based on patient data. For example, a user can input a patient presentation, including their age, gender, medical history, chief complaint, visual acuity, intraocular pressure, and optical coherence tomography (OCT) or fundus image interpretation and prompt the language model to provide a differential diagnosis (Table 1). However, a limitation is that ChatGPT can produce inaccurate results referred to as “hallucinations” [2]. When faced with a specialized topic, it may generate incorrect outputs due to insufficient depth and inaccurate details retrieved from the LLM’s database. More importantly, there is no guarantee that ChatGPT's suggestions are based on evidence-based guidelines or best practices. These drawbacks are clearly demonstrated in the response to the clinical prompt found in Table 1. Despite the patient data provided, with no significant medical history, diabetic macular edema was inaccurately given as the first diagnosis instead of the more fitting cystoid macular edema. In addition, the response inaccurately listed 45 years of age as a risk factor for age-related macular degeneration (AMD), a misleading assertion that could unnecessarily raise concerns. As it stands, the current iteration of ChatGPT, up to the point of this publication, contains knowledge acquired only from publicly available Internet text sources up until September 2021. Therefore, with the ability to “hallucinate" and its lack of recent data, ChatGPT should not be used as a sole source of clinical decision-making but rather a supportive tool for generating diagnoses and treatment plans.

Another clinical area in which the integration of ChatGPT presents a transformative opportunity is patient triage. Eye care professionals frequently grapple with managing a diverse range of patients, each presenting varying degrees of urgency. The incorporation of ChatGPT into the triage process offers a promising solution to this challenge. By engaging in an initial conversation with the artificial intelligence (AI), patients can effectively communicate their symptoms, allowing ChatGPT to assess the severity and urgency of their condition and direct them to the appropriate level of care. One notable limitation may be the potential for inaccuracies in the AI's assessment of patients' symptoms and conditions. Because the quality of ChatGPT's responses heavily depends on the accuracy, relevance, and representativeness of the data used for training, any shortcomings in the data quality may result in incorrect recommendations for the level of care needed. Furthermore, ophthalmology relies heavily on the physical exam, and ChatGPT does not have the ability to objectively examine and assess patients with current technological limitations.

Data security and Health Insurance Portability and Accountability Act (HIPAA) violations are other pressing concerns, as the use of ChatGPT might inadvertently involve sensitive patient information, including identities, medical histories, diagnoses, and treatments. Users must be aware that their input, which may contain sensitive data, could be integrated into the AI's response, potentially exposing confidential information. Moreover, if another user submits a similar prompt, ChatGPT may generate a response containing sensitive data from another user's prior interactions. Thus, diligently safeguarding this data from unauthorized access or misuse is essential.

Educational applications and limitations

ChatGPT's performance in answering ophthalmology exam questions has shown to be limited [4,5]. Antaki et al.'s recent study assessed ChatGPT's accuracy in answering multiple-choice questions from two well-known question banks used for the Ophthalmic Knowledge Assessment Program (OKAP) exam [4]. They found that ChatGPT's legacy model achieved 55.8% and 42.7% accuracy, while ChatGPT Plus reached 59.4% ± 0.6 and 49.2% ± 1.0 in the two simulated 260-question exams. Similarly, Mihalache et al. reported that ChatGPT scored 58% accuracy in multiple-choice questions and 54% accuracy in stand-alone questions [5]. These results suggest that ChatGPT may not have sufficient domain-specific knowledge or understanding of ophthalmic concepts and terminology to provide reliable answers or explanations to exam questions at this time.

Despite these limitations, the LLM can be a useful study aid for trainees and practitioners preparing for board certification exams by generating practice questions, explanations, and feedback. An example of this may involve a user prompting the LLM to write a board-style exam with answers and explanations on any topic of interest. For instance, a user could prompt: "Create a board-style question about the differential diagnosis and management of glaucoma, including the correct answer and an explanation for why that answer is correct." This way, users can tailor their study approach, focusing on areas they find challenging and receiving immediate feedback to enhance their understanding.

ChatGPT can also serve as a valuable educational tool in the realm of ophthalmology, particularly for patient education. Upon patient discharge, ChatGPT can generate tailored explanatory texts elucidating eye conditions, treatments, prescribed medications, and follow-up instructions in an easily comprehensible manner. The language used can be adapted to accommodate the patient's age, such as simplifying an amblyopia explanation for an 8-year-old (Table 1).

Similarly, ChatGPT can produce dialogues that emulate interactions between an ophthalmologist and patient. These dialogues may address common inquiries, concerns, and reassurances. Examples include elucidating cataract surgery risks and benefits, offering support in alleviating preoperative anxiety, and providing detailed postoperative care and follow-up guidance. As an illustration, a user could input: "I had cataract surgery last week and I'm experiencing some blurred vision and discomfort. Is this normal?" and the LLM might respond: "While some blurred vision can be normal after cataract surgery, it's important to report these symptoms to your doctor as they could indicate complications. I recommend you get in touch with your ophthalmologist as soon as possible." In contrast, a potential limitation of this application would be if ChatGPT overlooked a patient concern that would otherwise warrant further investigation. Consequently, these dialogues underscore the capacity of ChatGPT to provide immediate responses, potentially alleviating patients' fears and facilitating rapid response to potential medical concerns, while highlighting the continued necessity for professional medical oversight.

Research applications and limitations

The AI can be employed as a research assistant or collaborator, aiding in literature review, data analysis, manuscript development, and peer review processes. ChatGPT's capabilities include searching for pertinent articles, synthesizing key findings, generating hypotheses, conducting statistical analyses, composing abstracts or introductions, and offering constructive feedback. For instance, users can input a research question or keyword, and ChatGPT can furnish a list of recent publications, accompanied by summaries and citations, or provide descriptive statistics for an input dataset (Table 1).

**Table 1:** Ophthalmology-related prompts and responses from ChatGPT

	Clinical	Educational	Research
Prompt	What is the patient's differential diagnosis? A 45-year-old female with no significant medical history presented with a chief complaint of blurred vision in her left eye for the past week. Her visual acuity in the right eye was 20/20 and in the left eye was 20/50. Intraocular pressure was measured at 14 mmHg in the right eye and 16 mmHg in the left eye. OCT interpretation revealed thickening of the fovea with intraretinal cysts in the left eye. Fundus imaging showed a normal optic disc, but macular edema with hard exudates in the left eye.	Explain amblyopia for an 8-year-old	Conduct a literature review on the use of artificial intelligence in ophthalmology.
Response	This patient's differential diagnosis could include: Diabetic macular edema: This is a common cause of macular edema and can cause decreased vision in the affected eye. The patient's age and lack of medical history make this a possibility. Cystoid macular edema: This is another cause of macular edema that can be associated with a variety of underlying conditions, including inflammation and certain medications. Age-related macular degeneration (AMD): This is a condition that affects the macula and can cause blurred vision, distortion, or dark spots in the central vision. The patient's age is a risk factor for AMD. Retinal vein occlusion: This occurs when a blood clot or blockage forms in the veins that drain blood from the retina. It can cause blurred vision, and the patient's OCT findings of intraretinal cysts suggest this as a possibility. Macular telangiectasia: This is a rare condition that affects the blood vessels in the macula and can cause decreased vision and distortion. It may be a less likely possibility given the patient's lack of medical history. Further testing, such as fluorescein angiography or other imaging studies, may be necessary to make a definitive diagnosis and determine the appropriate treatment. Treatment options could include laser therapy, intravitreal injections, or other interventions depending on the underlying condition.	Amblyopia, also known as "lazy eye," is a condition where one eye doesn't see as well as the other eye. It happens when the brain and the eye are not working together as they should. Amblyopia can happen for different reasons, but one common reason is when one eye is weaker than the other eye. If the weaker eye is not used as much, the brain will start to ignore the signals it gets from that eye. This can make the weaker eye even weaker over time. To help treat amblyopia, doctors will often patch the stronger eye for a few hours each day. This forces the brain to use the weaker eye more, which can help it get stronger. Sometimes, eye drops or glasses can also help. It's important to treat amblyopia as early as possible, because the longer it goes untreated, the harder it is to fix. That's why it's important to have regular eye exams with an eye doctor, even if your eyes seem to be working well.	Artificial intelligence (AI) has been increasingly utilized in ophthalmology over the past several years. Here is a brief literature review on the use of AI in this field. One area where AI has shown promise is in the detection and diagnosis of diabetic retinopathy (DR). Several studies have demonstrated the feasibility and accuracy of using AI algorithms to detect DR from retinal images. For example, a study by Ting et al. (2017) developed an AI algorithm that was able to detect referable DR with a sensitivity of 90.3% and specificity of 91.1%. Similarly, a study by Gulshan et al. (2016) developed a deep learning algorithm that achieved a diagnostic accuracy comparable to that of human experts. AI has also been used to detect other retinal diseases, including age-related macular degeneration (AMD) and glaucoma. A study by Keel et al. (2019) used a deep learning algorithm to accurately detect AMD from color fundus photographs with an area under the curve (AUC) of 0.99. Another study by Li et al. (2019) developed a machine learning algorithm that was able to accurately classify glaucoma and healthy eyes from optical coherence tomography (OCT) images with an AUC of 0.96. In addition to diagnosis, AI has also been used to predict disease progression and treatment outcomes. A study by Asaoka et al. (2019) developed a machine learning algorithm that was able to predict visual field progression in glaucoma patients with an accuracy of 78.1%. Overall, AI has shown promise in ophthalmology for the detection, diagnosis, and prediction of disease progression. However, further studies are needed to validate the clinical utility and effectiveness of these algorithms in real-world settings. Additionally, ethical considerations around the use of AI in medicine need to be carefully addressed, including issues related to data privacy, algorithm transparency, and bias.

However, it is essential to acknowledge and address several limitations, including inaccuracies, algorithmic biases, data security concerns, and ethical considerations. As previously mentioned, the current iteration of ChatGPT is limited to the knowledge it acquired from publicly available Internet text sources up until September 2021. As a result, all requests that inquire the LLM on recent research may be outdated or inaccurate. Furthermore, AI systems like ChatGPT, if developed using datasets that do not adequately represent the diversity of the population, may exhibit algorithmic biases. This bias could potentially infiltrate healthcare-based machine learning systems, leading to skewed predictions of disease risk based on factors, such as race or gender, even when these factors are not actual causal agents. Thus, researchers must also verify AI-generated information and ensure that it is free from algorithmic biases, while also remaining vigilant about the quality and accuracy of the generated content. Ethical issues, such as plagiarism, data fabrication, and misrepresentation, must also be carefully considered to maintain research integrity. Researchers should thoroughly review and edit AI-generated content, ensuring proper citations and avoiding duplicate content. Ultimately, it is crucial to double-check any AI-generated data and not to become overly reliant on ChatGPT, preserving critical thinking and problem-solving abilities.

Conclusion

The use of ChatGPT in ophthalmology offers exciting potential, particularly in areas, such as clinical decision-making, education, and research, but it is not without limitations, including the risk of generating incorrect outputs and concerns over data security. Vigilance is paramount, especially in ensuring accuracy, addressing ethical considerations, and maintaining data privacy. As we journey into the era of AI in ophthalmology, the challenge lies in integrating these technologies in a manner that enhances patient care, while meticulously safeguarding accuracy and the welfare of our patients.
